# Application of COSMO-RS-DARE as a Tool for Testing Consistency of Solubility Data: Case of Coumarin in Neat Alcohols

**DOI:** 10.3390/molecules27165274

**Published:** 2022-08-18

**Authors:** Piotr Cysewski, Tomasz Jeliński, Maciej Przybyłek

**Affiliations:** Department of Physical Chemistry, Pharmacy Faculty, Collegium Medicum of Bydgoszcz, Nicolaus Copernicus University in Toruń, Kurpińskiego 5, 85-950 Bydgoszcz, Poland

**Keywords:** coumarin, solubility, COSMO-RS, COSMO-RS-DARE, intermolecular interactions, aliphatic alcohols, neat solvents

## Abstract

Coumarin is a naturally occurring lactone-type benzopyrone with various applications in the pharmaceutical, food, perfume, and cosmetics industries. This hydrophobic compound is poorly soluble in water but dissolves well in protic organic solvents such as alcohols. Despite the extensive use of coumarin, there are only a few reports documenting its solubility in organic solvents, and some reported data are incongruent, which was the direct impulse for this study. To resolve this problem, a theoretical congruency test was formulated using COSMO-RS-DARE for the determination of intermolecular interaction parameters, which allowed for the identification of outliers as suspicious datasets. The perfect match between back-computed values of coumarin solubility and the experimental ones confirms the reliability of the formulated theoretical approach and its adequacy for testing solubility data consistency. As the final approval, the temperature-related coumarin solubility in seven neat alcohols was determined experimentally. Four solvents (methanol, ethanol, 1-propanol, and 2-propanol) were used for reproducibility purposes, and an additional three (1-butanol, 1-pentanol, and 1-octanol) were used to extend the information on the homologous series. The consistency of this extended solubility dataset is discussed in terms of the comparison of remeasured solubility values with the ones already published and within the series of structurally similar solvents. The proposed procedure extends the range of applicability of COSMO-RS-DARE and provides a real and useful tool for consistency tests of already published solubility data, allowing for the approval/disapproval of existing data and filling gaps in datasets. Linear regressions utilizing a 2D molecular descriptor, SpMin2_Bhm, or the distance between solute and solvent in the Hansen solubility space, R_a_, were formulated for the estimation of COMSO-RS-DARE integration parameters.

## 1. Introduction

Solubility values are one of the most fundamental physicochemical properties of organic solids, including active pharmaceutical ingredients (APIs) [1,2], and such data have both practical and theoretical importance. Solvents are used at multiple stages of the API manufacturing process, including extraction, synthesis, crystallization, and purification. All of these steps rely on the dissolution behavior of pharmaceuticals and their intermediates. Obtaining bioactive compounds from natural sources, including plants, algae, and biowastes, is often the first step requiring the usage of an appropriate solvent. The most suitable solvents are characterized by a good extraction potential, selectiveness, and environmental friendliness [3,4,5,6]. For example, the common method of purification of synthesized or natural APIs is crystallization, and for this purpose either a good solubilizer has to be selected or the optimal solvent/anti-solvent combination [7,8].

Coumarin (C_9_H_6_O_2_, 2H-1-benzopyran-2-one, CAS 91-64-5) is a chemical compound that is wide-spread in nature, occurring, for example, in tonka beans, cinnamon, and vanilla [9,10,11]. It has found applications in many areas, including dye-sensitized solar cells [12], fluorescent probes [13], sensitizers [14], laser dyes [15], and inhibitor additives to different materials [16]. Even more importantly, coumarin and its derivatives have some beneficial biological activities, including antioxidant [17], anticoagulant [18], antibacterial [19], anti-inflammatory [20], antiviral [21], and enzyme inhibitory [22] actions, which make them widely used in medicine [23].

Considering coumarin manufacturing, solvent selection plays a crucial role. Aliphatic alcohols, especially biobased ones such as ethanol, glycerol, and 2-propanol, are considered to be efficient and relatively green extracting agents for nutraceuticals [24,25]. For instance, according to Bourgaud et al. [26], proton-donating and polar solvents, such as water, ethanol, and methanol, were more efficient for the extraction of coumarin from *Melilotus officinalis* L. than chloroform, diethyl ether, and ethyl acetate. This is understandable since coumarin and its derivatives are, in general, highly proton-accepting molecules [27,28,29]. Furthermore, the aqueous solubility of coumarin decreases with the increase of pH [30], which confirms its relatively basic character.

The nature of solute and solvent, determining their mutual affinity, and the thermodynamic parameters related to the stability of the crystal lattice, are the key factors affecting solubility. Among a variety of theoretical models developed to describe the solubility–temperature relationships, the in silico quantum-chemical computations are the most intriguing since they are based on the information retrieved from the 3D molecular structure. In principle, they provide actual solubility predictions [31], in contrast to many models offering merely back-computations, such as, for example, the van’t Hoff [32], Apelblat [33,34], Buchowski-Ksiazczak (λh) [35], Wilson [36], NRTL [37], and Jouyban-Acree [38] models. One of the most recognized in silico approaches is the conductor-like screening model for real solvents, COSMO-RS [39,40,41,42]. It deserves special attention due to its efficiency, universality, and clear thermodynamic interpretation and for following chemical intuition.

The aim of this paper is fourfold. First of all, the theoretical protocol for resolving inconsistencies in reported solubility data is formulated. Second, a validation of this theoretical approach is performed by experimental remeasurements of already published solubility data. Third, for providing a broader perspective, a series of structurally similar solvents are used for additional confirmation of the theoretically drawn conclusions about the coherency of the solubility dataset by including newly measured cases. Finally, an interpretation of the COSMO-RS-DARE parameters is offered by correlating the computed values with popular molecular descriptors.

## 2. Materials and Methods

### 2.1. Materials

Coumarin (CAS: 91-64-5, MW = 143.14 g/mol) was purchased from Sigma Aldrich (Saint Louis, MO, USA), and the purity of this chemical was ≥99% according to the supplier. All of the solvents used were also provided by Sigma Aldrich and included: methanol (CAS: 67-56-1, MW = 32.04), ethanol (CAS: 64-17-5, MW = 46.07 g/mol), 1-propanol (CAS: 71-23-8, MW = 60.10 g/mol), 2-propanol (CAS: 67-63-0, MW = 60.10 g/mol), 1-butanol (CAS: 71-36-13, MW = 74.12), 1-pentanol (CAS: 71-41-0, MW = 88.15 g/mol), and 1-octanol (CAS: 111-87-5, MW = 130.23 g/mol). The purity of all solvents was ≥99%.

### 2.2. Calibration Curve

The calibration curve used for the determination of coumarin solubility was prepared with the use of a stock solution of coumarin in methanol with a concentration of 0.0333 mg/mL. This solution was then successfully diluted by transferring its fixed amounts to 10 mL volumetric flasks and adding methanol. The obtained series of solutions with decreasing coumarin concentrations was measured spectrophotometrically, and the absorbance values found at 310 nm wavelength were plotted against the corresponding concentration values. Three separate curves were prepared and then averaged, with the resulting linear equation being y = 42.346x + 0.0085. The curve can be characterized by high linearity, with the determination coefficient of R^2^ = 0.9997. The limit of detection (LOD) and limit of quantification (LOQ) were found to be LOD = 4.93 × 10^−4^ mg/mL and LOQ = 1.48 × 10^−3^ mg/mL, which were well below the coumarin concentrations found in the samples.

### 2.3. Sample Preparation

In this study, the shake-flask-type method of solubility determination was used. As documented in previous studies [43,44,45,46,47,48], the applied procedure was found to be useful and reliable in the case of various pharmaceutically active compounds. The samples used for solubility measurements were prepared by adding an excess amount of coumarin to 10 mL volumetric flasks that were then filled with the appropriate solvent in order to obtain saturated solutions. The prepared samples were incubated for 24 h at four different temperatures (25–40 °C with 5 °C intervals) using an ES-20/60 Orbital Shaker Incubator supplied by Biosan (Riga, Latvia). The temperature adjustment accuracy was 0.1 °C, and its variance during the daily cycle was ±0.5 °C. Moreover, the mixing of the samples at 60 rev/min was provided by the incubator. In the next step, the samples were filtered using a syringe with a PTFE filter with a 22 µm pore size. All of the test tubes, syringes, and filters were preheated to the appropriate temperature to avoid the precipitation of dissolved coumarin. Finally, fixed amounts of the filtrate were transferred to test tubes, which were prefilled with a set amount of methanol, and samples prepared in this way were measured spectrophotometrically.

### 2.4. Solubility Measurements

The concentration of coumarin in the samples was determined through spectrophotometric measurements using an A360 spectrophotometer from AOE Instruments (Shanghai, China). Spectra in the 190–700 nm wavelength range were recorded with a 1 nm resolution, and samples were diluted with methanol, which was also used as a reference. The absorbance values at 310 nm were used in conjunction with the calibration curve prepared earlier. Three samples were measured for each data point, and their concentrations were averaged and expressed as mole fractions.

### 2.5. COSMO-RS-DARE Computations

COSMO-RS-DARE (dimerization, aggregation, and reaction extension) [49] is an adaptation of the COSMO-RS approach [39,40,41] for treating bulk systems by including concentration-dependent composition alterations imposed by diverse reactions in the bulk system. The adaptation of this approach for solids solubility computations was detailed in our previous papers dealing with neat solvents [50], binary mixtures [45], and ternary systems [51]. Therefore, only brief remarks are provided here. In this study, both COSMO-RS and COSMO-RS-DARE computations were carried out using COSMOtherm (version 22.0.0, Dassault Systèmes, Biovia: San Diego, CA, USA) [52] software. The most important difference between these approaches is the definition of conformers included in the thermodynamic property computations. In both methods, each component is represented by the set of the most representative conformers, which are identified during the initial phase prior to the actual thermodynamic property computations. Typically, the conformational analysis leads to a set of low-energy structures of every component of the considered bulk system. For this purpose, the COSMOconf program [53] can be used to offer an automatization of this process. However, such a procedure is unable to identify multicomponent clusters that might potentially occur in the system due to direct interactions between components. These complexes can significantly differ in their electron density properties, which are essential for the COSMO-RS approach. Hence, the pool of conformers of monomers is extended, with the ones identified in the separate conformational analysis including bimolecular contacts. After generating potentially important pairs, the full clustering algorithm is applied to reduce the number of conformers and eliminate very similar clusters or those of low probability due to the energy values exceeding the selected threshold with respect to the most favorable one. All the results presented here were computed using the TURBOMOLE program [54,55] with in-house-developed scripts. There is one important modification implemented in this paper due to problems encountered during the optimization of coumarin dimers in the case of applying the RI-DFT BP86 (B88-VWN-P86) functional along with the def-TZVP basis set. For consistency with parametrization sets of COSMOtherm, the geometries optimized in such a manner are used for the final generation of “cosmo” and “energy” files by single-point energy computations in an extended def2-TZVPD basis set using the same functional. Unfortunately, optimization trials of coumarin pairs failed using this approach since coumarin, as an aprotic compound, cannot form dimers stabilized by hydrogen bonds. Pairs of this type were identified in the case of heteromolecular contacts with proton-donating molecules such as alcohols or water. However, the expected stacked dimers of coumarin were not obtained since monomers tend to separate from each other while using this type of geometry optimization. The reason for this was the inappropriate accounting of contributions coming from electron dispersion and non-covalent interactions. Hence, geometry optimization was performed using an alternative approach. Since the basis superposition error (BSSE) can be an important contribution to pairs stabilization energy, the geometry optimizations were performed in consistency with geometry-based counterpoise corrections (gCP) [56] of the pairs stabilization energies. This parametrization relies on the BP97 GGA functional used with the def2-SVPD basis set and Grimme D3-BJ dispersion corrections [57]. Fortunately, this level of geometry optimization led to stable coumarin pairs in stacked conformations, which is not surprising since this meta-GGA functional is commonly accepted as a reliable one for intermolecular interaction characteristics [58]. Hence, the obtained geometries of pairs were used for the preparation of the final input files, which were necessary for the utilization of the BP_TZVPD_FINE_22.ctd parametrization in COSMOtherm by completing the single-point energy computations on the required RI-DFT BP86/def2-TZVPD level.

For every system, the COSMO-RS-DARE approach introduces two fitting parameters defining intermolecular interactions between those centers that are not included in the default COSMO-RS statistical analysis. The mathematical form describing these parameters resembles the Gibbs free energy equation as follows:

(1) $$G_{ij}^{\mathrm{int}}\left(T\right)=H_{ij}^{\mathrm{int}}-T\cdot S_{ij}^{\mathrm{int}}$$

where the enthalpic, $H_{ij}^{\mathrm{int}},$ and entropic, $S_{ij}^{\mathrm{int}},$ contributions of the interactions between the *i*-th and *j*-th species are considered to be temperature-independent. These parameters are computed by fitting to experimental solubility data. It is important to note that the linear function defined by Equation (1) holds for many systems with very high accuracy, irrespective of the number of temperatures used for solubility determination. The intermolecular interaction thermodynamic parameters defined in Equation (1) can be used for assessments of enthalpy–entropy compensation by the analogy to a similar analysis conducted with the aid of a solvation thermodynamic function determined via a van’t Hoff plot. Here, the following definition is the direct analogy of the enthalpy–entropy compensation factor:

(2) $$\chi =\frac{\left[H_{ij}^{\mathrm{int}}\right]}{\left[H_{ij}^{\mathrm{int}}]+[T\cdot S_{ij}^{\mathrm{int}}\right]}$$

The meaning remains the same as in the original formulation and simply quantifies the contributions of the energetic and entropic terms in the total Gibbs free energy of interaction. Indeed, the dominance of the energetic component of the interaction in the system is indicated by values higher than 0.5. In the case of values lower than half, the entropic contribution overrides the enthalpic one.

## 3. Results and Discussion

The direct impulse for this work was the observed incongruences in the reported solubility data of coumarin provided by Huang et al. [59,60] (set A) and Akay et al. [61] (set B). Particular interest was narrowed to the alcohols used as neat solvents. The published values differ dramatically, especially for elevated temperatures. This problem was already raised by Akay et al. [61], who honestly provided data comparison. The authors offered general explanations for the observed inconsistencies, relating them mainly to the accuracy of the analytical method or solution–solid equilibrium establishment. However, the scientific audience was left with two distinct sets of solubility data. The observed deviations of the reported values most probably cannot characterize the same physical system, and some serious methodological issues are at play. Although it is difficult to compare different datasets due to the different experimental conditions, according to Huang et al. [59,60], the solution–solid equilibrium in the case of coumarin can be achieved after 8 h, which includes 3 h without agitation for the settling of undissolved particles. In the case of Akay et al. [61], the sample agitation and incubation time was 18 h. It is noteworthy that in this study the samples were equilibrated for an even longer period (24 h).

Since solubility data are used for very detailed thermodynamic analysis, conflicting values lead to completely different system characteristics. It is then of crucial importance to critically assess the solubility data, especially since they directly affect the validity of the preferential solvation analysis performed [61] in terms of the inverse Kirkwood–Buff integrals formalism. The most straightforward way, of course, would be a repetition of the experiments with the hope that new measurements would resolve the observed conflict, including eventual recomputations of coumarin-preferential solvation. However, without avoiding this goal, the motivation of this work is a bit wider, aiming to develop a general theoretical approach that might be used for testing solubility data consistency. Coumarin is merely an exemplary case that is used for validation purposes and to direct further experiments. In our local solubility database, there are many more such cases, and their identification for data curation might be interesting for a broad scientific audience. In this context, it is worth citing at this point a precedent that took place in the circumstance of the critical evaluation of maleic acid solubility [62]. Hence, a theoretical tool suitable for data consistency analysis seems to be valuable.

### 3.1. The COSMO-RS-DARE Solubility Computations

**Figure 1:**
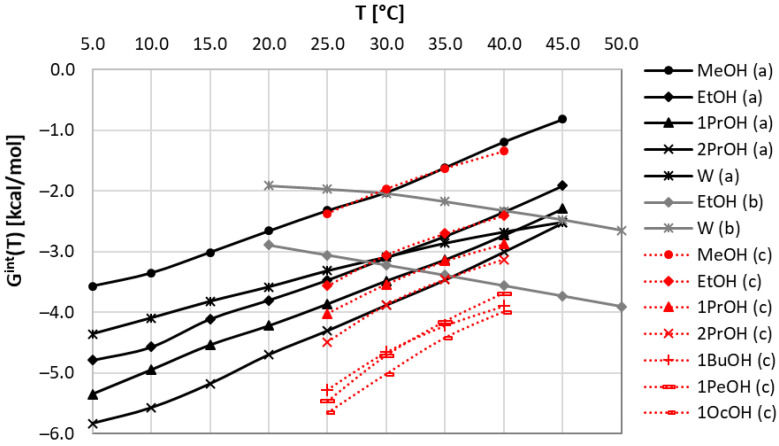
Temperature trends of COSMO-RS-DARE interaction parameter, G^int^(T), characterizing coumarin solubility in neat alcohols and water. Black solid lines represent set a [59,60], grey solid lines correspond to set b [61], and red dotted lines characterize data measured in this work (set c).

There are several first-principle approaches that might be adopted for the purpose of consistency tests of available solubility data. From our perspective, the method of first choice is the COSMO-RS framework [39,42], which is often applied for characteristics of bulk systems using only information about molecular structure. Unfortunately, solubility computations using this method suffer, in many cases, from serious inaccuracies [44,45,50], although some systems can be characterized with quite satisfactory correctness [43,50]. Previously published reports [44,45,51] suggested that one possible way is to enhance the default COSMO-RS computation with the inclusion of information about intermolecular interactions occurring in the saturated systems by providing the structures of binary clusters. This is carried out via the COSMO-RS-DARE framework, formulated as an extension of COSMO-RS for modeling systems showing concentration-dependent alterations of qualitative and quantitative compositions. As mentioned in the Methods Section, this approach offers very high accuracy at a cost of two fitting parameters for every system. However, these parameters have a deeper meaning, and the origin of their introduction is also quite clear. Indeed, the proper definition of a solute–solvent system requires an adequate representation of solute conformations. This includes not only the monomeric forms of flexible compounds but also structures altered by direct contacts with the constituents of the bulk system. In the case of neat solvents, two types of such clusters can play the most important role, namely, solute–solute and solute–solvent interactions. The third type, which is the solute–solute interaction, is ignored in the case of solubility computations [44,45]. It was already documented [63] that the restriction to include just pairs is quite sufficient for a proper representation of intermolecular clusters in the solubility computations. The same approach is applied here for the determination of values of these two fitting parameters defined by Equation (1). The obtained values characterizing set A and set B are collected in Table 1. In addition, in Figure 1, the temperature trends of corresponding G^int^ values are plotted for these systems. First of all, it is worth emphasizing that, according to expectations, a high linearity of all plots is observed. This is a fortunate circumstance justifying the computation of only two parameters per system. Furthermore, one can see that discrepancies in the solubility values between the two considered sets of data seriously affect the intermolecular interaction parameter, G^int^(T). There are observed opposite trends of all black and gray lines, suggesting reverse temperature trends. The interactions in set A decrease with the rise of temperature, whereas for set B an opposite conclusion is drawn. This is associated with the discrepancies in the obtained H^int^ and S^int^ data, as provided in Table 1. In the case of water and ethanol, the values of H^int^ are positive for set B, contrary to the values obtained for set A. A similar change in the sign is observed for the entropic contribution, S^int^.

**Figure 2:**
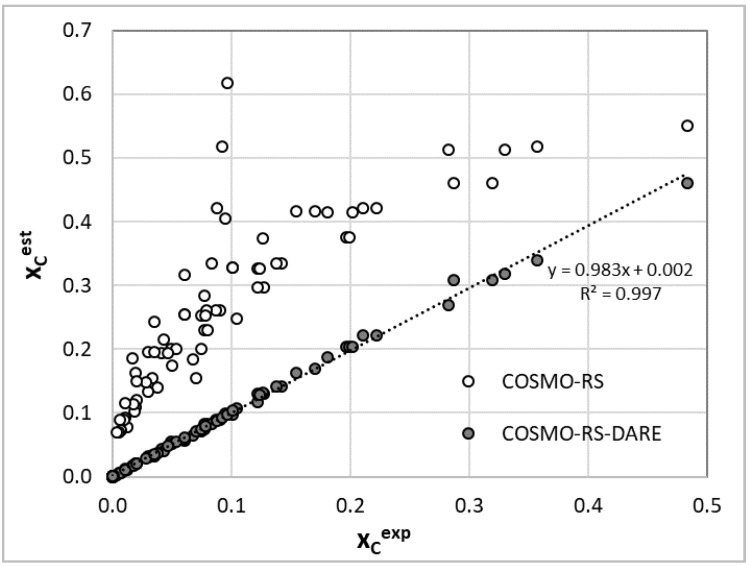
Correlation between estimated and experimental solubility of coumarin in neat alcohols and pure water.

In conclusion to this part, it is worth emphasizing that the application of COSMO-RS-DARE for the determination of interaction parameters clearly distinguishes set A from set B. Although there seems to be an internal consistency within each of these sets, the comparison of solubility with data measured by Huang et al. [59,60] in structurally similar solvents suggests the consistency of set A with the general trends of the computed values of G^int^(T). Hence, based on this observation, one should cautiously accept that set A more reliably characterizes coumarin solubility in selected alcohols. To further support this conclusion, it is worth inspecting the accuracy of the performed first-principle solubility computations. The correlation between the estimated and experimental values is presented in Figure 2 and suggests that the COMSO-RS-DARE predictions are very accurate, in contrast to the default COSMO-RS predictions. Hence, the high reliability of the COSMO-RS-DARE computations of solubility in neat solvents supports the application of this approach for the inspection of dataset consistency.

### 3.2. The Experimental Validation of Theoretical Consistency Test

**Figure 3:**
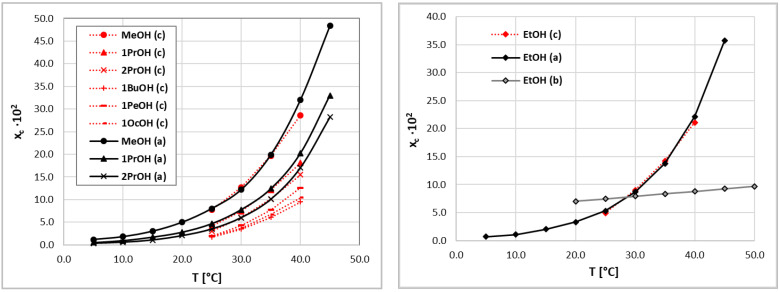
Solubility values of coumarin measured in pure alcohols. The notation is consistent with the one used in Figure 1.

The final approval of the considered method must always come from experiments. Hence, the validity of the consistency test of coumarin solubility data performed in the theoretical manner was inspected by performing a sequence of new experiments for a series of seven alcohols. Four were used to directly reproduce the solvents belonging to set A, and in addition, three new solvents were included to extend the set of structurally similar solvents. The obtained values are plotted in Figure 3, and numerical data are collected in the Supplementary Materials (Table S1). In the left panel of Figure 3, the results of the mole fraction solubility of coumarin in neat alcohols, except for ethanol, are collected. The coumarin solubility in this solvent is plotted in the right panel of Figure 3, along with the one belonging to set B [61]. The presented results clearly confirm the observations made due to the performed COSMO-RS-DARE analysis. The results of our measurements of coumarin solubility in ethanol are almost identical to those included in set A, with very similar temperature solubility trends. Although the results of our measurements tend to be slightly lower for the more elevated temperatures compared to the data provided by Huang et al. [59,60], the error is within a few percent. The same conclusion can be drawn from the analysis of the rest of considered systems.

The COSMO-RS-DARE was also applied for the interpretation of the new solubility data reported in this work, and the resulting intermolecular parameters are collected in Figure 1 and Table 1, documenting a coherent pattern of our measurements and those included in set A [59,60]. However, the mentioned slight deviation between these two sets at higher temperatures also results in slightly lower values of H^int^ and S^int^, as documented in Table 1.

**Figure 4:**
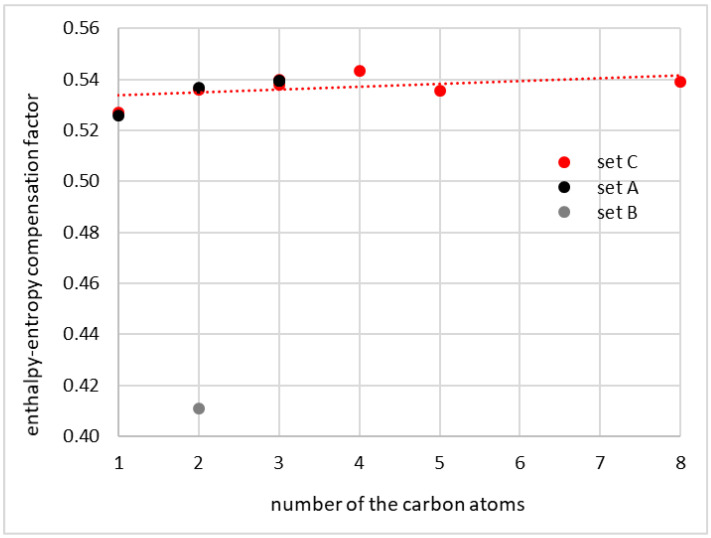
The correlation between the enthalpy–entropy compensation factor and the number of carbon atoms in alcohol molecules computed for room temperature.

For the final test of the consistency of the coumarin solubility data, the values of the enthalpy–entropy compensation factor, χ, were determined for all considered series. In the solubility literature, this parameter is used in the context of apparent solvation enthalpy and entropy determined using the van’t Hoff model. Here, formally, the same formula is applied, but the thermodynamic values are represented by interaction parameters determined using COSMO-RS-DARE. Hence, the values collected in Table 1 and plotted in Figure 4 were computed using Equation (2) for T = 298.15 K. A modest linear trend of χ in relation to the number of carbon atoms in the solvent molecules of neat alcohols is observed. This allows for at least qualitative characteristics and is supposed to be sufficient for the identification of outliers. Indeed, the values of χ computed for sets A and C suggest that the energetic contribution has a dominant effect on the total Gibbs free energy of interaction, and the entropic term computed for room temperature reaches roughly one third of the interaction energetics. Conclusions drawn from the data of set B are exactly the opposite, suggesting stronger contributions of the entropic term, which seems to be unlikely.

### 3.3. COSMO-RS-DARE Parameters Interpretation

**Figure 5:**
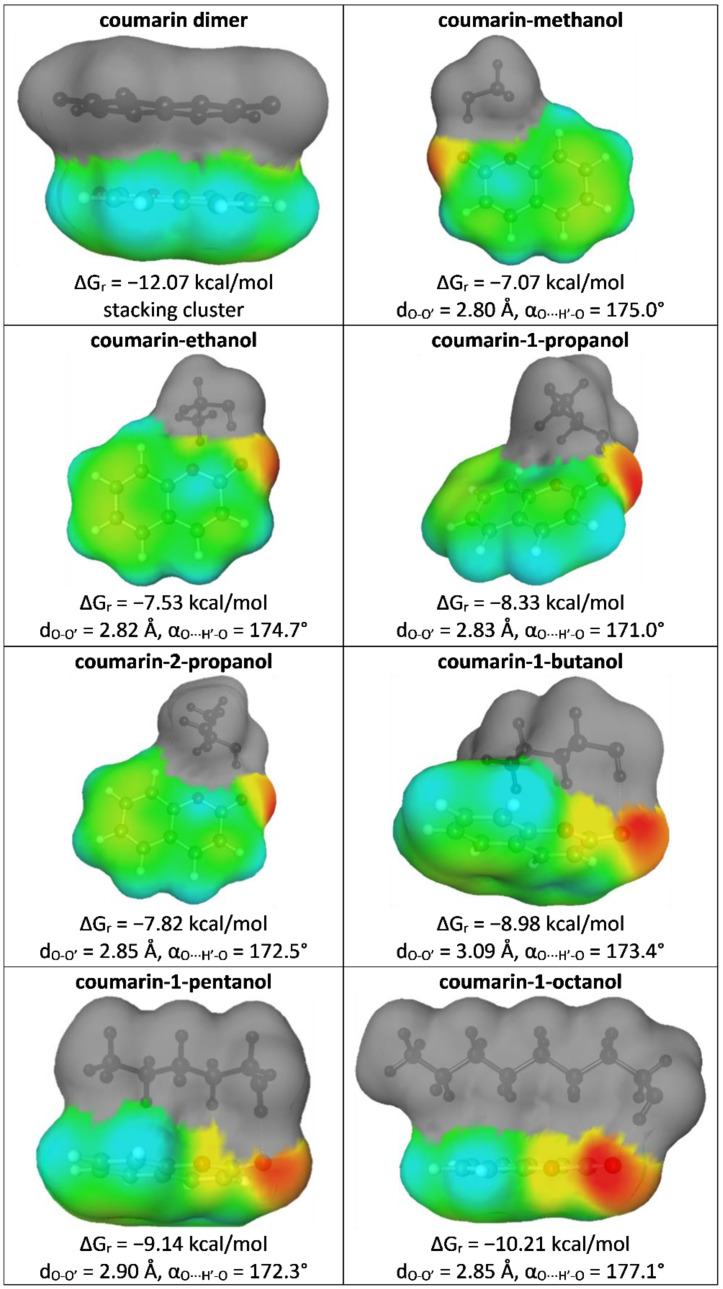
Characteristics of selected energetic and geometric properties of the most stable pairs of coumarin with constituents of the studied systems.

Although this paper was prepared with a very simple and practical aim, which is solubility data curation, the application of COSMO-RS-DARE offers many interesting details regarding the mechanism of solubility. Hence, some additional information is provided here for the enhancement of the solubility interpretation of coumarin in neat alcohols. Using this sophisticated methodology requires prior generation, optimization, and clustering of pairs formed between the solute and solvent molecules. The most stable clusters are collected in Figure 5, along with energetic and geometric characteristics.

It is interesting to note that based on the data provided in Figure 5 it can be concluded that the elongation of the aliphatic chain affects the mutual orientation of the interacting alcohol molecules with respect to coumarin, but it only slightly alters the geometry of the hydrogen bond. The more carbon atoms in the alcohol molecule, the stronger the contribution of dispersion interactions and the overlapping of interacting molecules. Indeed, methanol is exposed outside and is placed in the plane with respect to coumarin. Starting from butanol, the alcohol molecules were placed above the plane of coumarin, enabling interactions of aliphatic chains with coumarin rings. Coumarin is highly aromatic, which is granted by the six-carbon ring. Hence, long enough chains are strongly attracted by delocalized electrons, which is responsible for strengthening the stability of intermolecular interactions, as documented by the values of the Gibbs free energy of reaction, leading to given pairs in the given solution at room temperature.

**Figure 6:**
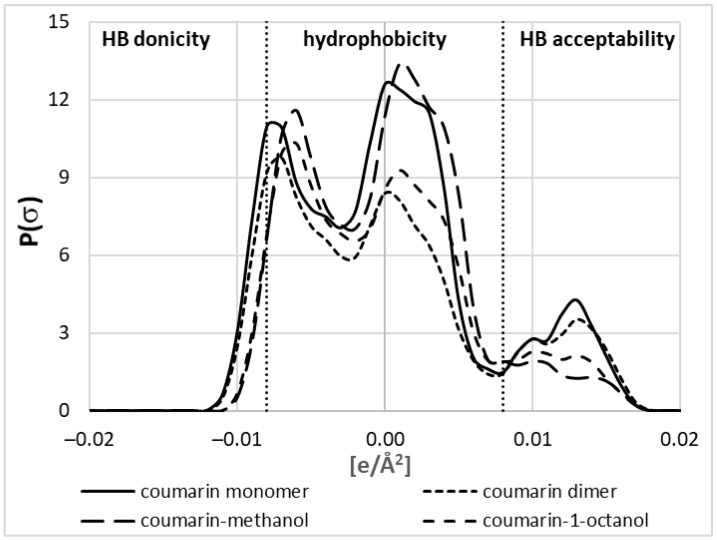
Exemplary σ-profile plots characterizing the coumarin monomer not involved in direct contacts, the dimer in stacked conformation, and hydrogen-bonded heteromolecular pairs with methanol and 1-octanol. The hydrophobicity region was defined as the charge density range between −0.08 and +0.08 e·Å^−2^.

The application of COSMO-RS-DARE has one serious disadvantage, which is the necessity to introduce two fitting parameters accounting for interactions between new pseudo-conformers constituting solute–solvent complexes. The first question is whether these new species are different from monomeric conformers. The answer to this question is provided in Figure 6, which collects σ-profiles computed for the coumarin monomer and molecules involved in pair formation. The σ-profiles, which have a simple meaning of histograms collecting distributions of charge density, are in line with chemical intuition. As depicted in Figure 6, three regions can be distinguished, characterizing hydrogen bonding properties and hydrophobicity. The comparison of the plots provided for coumarin monomer, dimer, and pairs with methanol or octanol suggests that intermolecular interactions seriously affect charge density distributions.

According to expectations, the HB donicity of coumarin is negligible irrespective of the form (monomeric or dimeric). A slightly higher HB acceptability of coumarin is expected due to the presence of electronegative centers. However, the strong aromaticity of coumarin is associated with its high hydrophobic character. It is interesting to note that hydrophobic interactions are seriously affected by the intermolecular interactions of coumarin. Indeed, dimers adopting stacked conformations are significantly less hydrophobic compared to the free monomer. Moreover, the interactions with long-chain alcohols reduce the hydrophobicity of such coumarin conformers due to the abovementioned structure of such heteromolecular complexes. On the contrary, interactions with methanol increase the hydrophobicity of coumarin due to the induction effect being the consequence of hydrogen bond formation. Furthermore, there is also a visible influence of the hydrogen bond formation on the HB acceptability of coumarin involved in pairs. It is quite understandable that interaction with methanol depletes its acceptability for further hydrogen bonding. On the other hand, dimers are as much prone to HB formation as monomers. There is also an interesting trend of HB donicity with affinities of coumarin to alcohols. The higher the ΔG_r_ (lower affinity), the stronger the alteration of HB acceptability. Hence, conclusions drawn from the exemplary σ-profiles provided in Figure 6 emphasize the necessity of including new species of coumarin in the description of saturated solutions in alcohols, and omitting them is one of the sources of the failed solubility computations of the default COSMO-RS approach. 

However, a problem remains with the parameters introduced by COSMO-RS-DARE, the values of which are to be derived based on experimental data. It is reasonable to expect that some external characteristics might help in estimating the values of these interaction parameters. One such example was already provided in the case of ethenzamide [50], for which a linear relationship was found between the COSMO-RS-DARE parameters and pairs affinity. The general problem with finding relationships of this type is the interplay of the shortcomings of the COSMO-RS model and the improper systems definition in the solubility computations. For chemically different solutes dissolved in solvents of different physicochemical properties, these two contributions are not equal, making it harder to find molecular descriptors suitable for accounting for the values of G_int_.

However, the situation is not as hopeless as it might seem. Here, a structurally similar set of solvents is studied with the same solute. In such a restricted chemical space, finding appropriate molecular descriptors for G^int^ computation seems to be feasible. To demonstrate this possibility, several descriptors were computed using the PaDEL software [64] and related to the interaction parameters resulting from the COSMO-RS-DARE computations. Additionally, the Hansen solubility parameters (HSP) expressing dispersion (δ_d_), dipolar (δ_p_), and hydrogen-bonding forces (δ_HB_) were retrieved from *Hansen Solubility Parameters: A User’s Handbook* [65] and used for the determination of the solubility space distance (R_a_) values between coumarin and alcohols (Equation (3)).

(3) $$R_{a}=\sqrt{4{\left(\delta_{d}^{solute}-\delta_{d}^{solvent}\right)}^{2}+{\left(\delta_{p}^{solute}-\delta_{p}^{solvent}\right)}^{2}+{\left(\delta_{HB}^{solute}-\delta_{HB}^{solvent}\right)}^{2}}$$

Although the HSP concept is very popular in polymer studies, it has also been widely applied for describing the solubility behavior of low-molecular-weight compounds in popular neat and multicomponent organic solvents, including aliphatic alcohols [66,67,68,69,70,71]. It is noteworthy that HSP were also applied in the case of coumarin for describing the solubilizing effects of choline-based natural deep eutectic solvents [72]. Therefore, HSP and R_a_ were included in the set of descriptors considered in this study.

**Figure 7:**
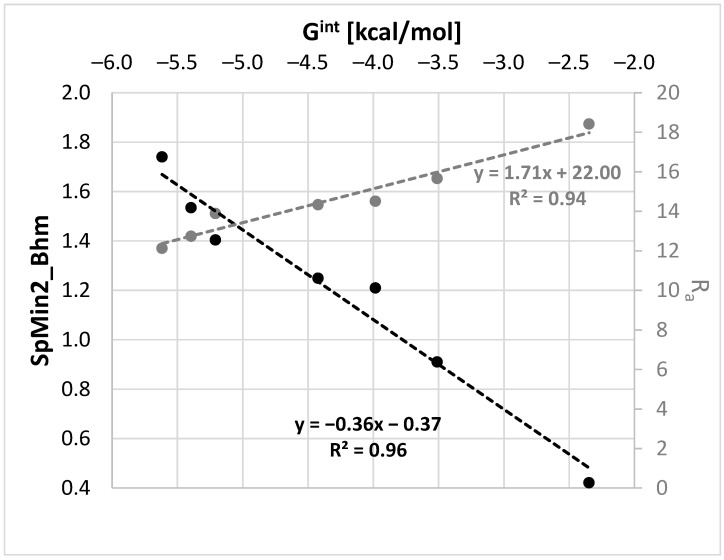
Exemplary regressions documenting the quality of the linear relationships between the COSMO-RS-DARE interaction parameter, G^int^, and molecular descriptors SpMin2_Bhm (Burden modified eigenvalue descriptor) and Ra (distance between solute and solvent in the Hansen solubility space).

Since G^int^ is temperature-dependent and the above molecular descriptors are not, the linear regressions were defined separately for each temperature used in our measurements. In Figure 7, the regression plots for room temperature are exemplified.

**Figure 8:**
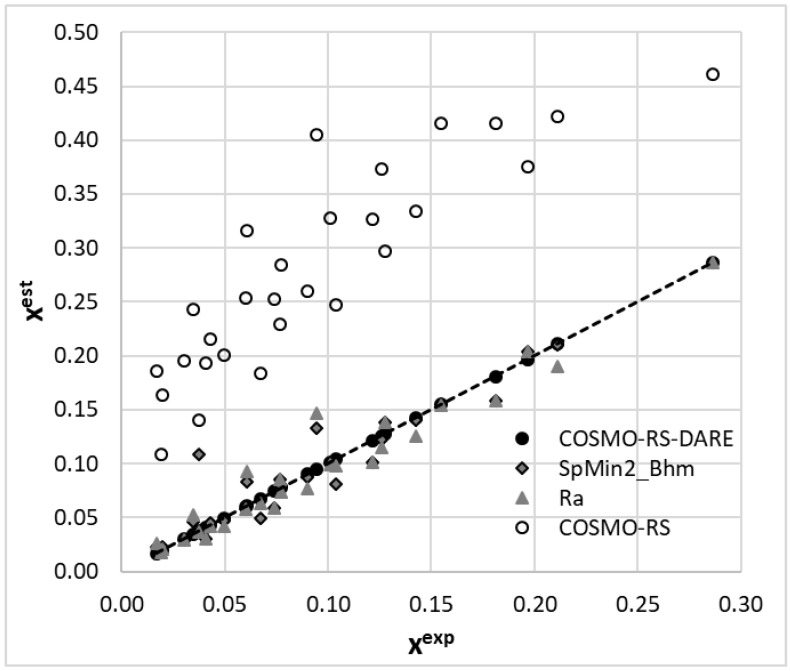
The results of the solubility prediction of coumarin in neat alcohols using the COSMO-RS and COSMO-RS-DARE approaches. The back-computed solubility values with G^int^ parameters fully fitted to experimental data were augmented by the ones resulting from the linear relationships provided in Figure 7.

Among many molecular descriptors computable in PaDEL, the Burden modified eigenvalue descriptor, SpMin2_Bhm, was found to be linearly correlated with G^int^. This is a relative mass weighted 2D molecular index related to the smallest absolute eigenvalue of a Burden modified matrix [73,74,75]. Since, there is a very good correlation between SpMin2_Bhm and δ_p_ (R^2^ = 0.94) or δ_HB_ (R^2^ = 0.97), the physical meaning of this parameter is associated with the polarity and hydrogen bond formation abilities of the solvent. As evidenced in Figure 7, the values of the distance between the solute and solvent in the Hansen solubility space can also be used as a measure of G^int^. This fortunate circumstance of a linear relationship enables computations of G^int^, which might be further used in COMSO-RS-DARE calculations. The results of such computations are provided in Figure 8, which also includes the results of the default solubility predictions. It is worth mentioning that the models are statistically meaningful. The values of the SpMin2_Bhm and R_a_ descriptors used for the G^int^ calculation and the statistical characteristics of the regression between x^est^ and x^exp^ are provided in the Supplementary Materials (Tables S2 and S3).

The mean average percentage error of the solubility values computed using G^int^ estimated with the aid of the SpMin2_Bhm descriptor was 15.2% (RMSD = 0.021), whereas the analogical value corresponding to R_a_ was equal to 14.0% (RMSD = 0.016). This is less precise compared to the G^int^ values fully fitted to the experimental data but much higher if confronted with a 240% mean average percentage error (RMSD = 0.19) for the default COSMO-RS computations of coumarin solubility.

## 4. Conclusions

It was already demonstrated in previous reports that COSMO-RS-DARE offers a substantial improvement in solubility computations over default COSMO-RS predictions. Although it requires experimental data for finding the values of its parameters by a fitting procedure, the linear dependence between the enthalpic and entropic contributions of interaction Gibbs free energy enables using just two parameters for very accurate back-computations of experimental solubility data. In this report, a new range of applications of COMOS-RS-DARE is demonstrated. The observed discrepancies in the reported solubility of coumarin in ethanol were resolved based on the temperature trends of interaction Gibbs free energy values. Outliers can be clearly identified based on interaction parameters, either by the inspection of G^int^(T) temperature trends or the values of temperature-independent components, H^int^ and S^int^. Importantly, G^int^ was found to be correlated with popular molecular features related to polarity and hydrogen-bonding forces, which is consistent with chemical intuition. A univocal validation of this approach was conducted by providing the results of new measurements of coumarin in neat alcohols. Four solvents were selected for the direct duplication of molar fraction solubility under saturated conditions, and an additional three were considered for the inspection of the broader trend within the aliphatic alcohol series. The consistency of the data was discussed, not only in terms of the comparison of the remeasured solubility data with those already published but also within the series of structurally similar solvents. The proposed procedure not only extends the range of applicability of COSMO-RS-DARE but provides a real and useful tool for consistency tests of already published solubility data. This can be a valuable guide for planning solubility measurements, reducing experimental efforts for data duplication or verification and building a coherent solubility database. The procedure can be easily extended to multicomponent mixtures of solvents. 

## Figures and Tables

**Table 1:** Results of COSMO-RS-DARE computations characterizing coumarin solubility in neat methanol (MeOH), ethanol (EtOH), 1-propanol (1PrOH), 2-propanol (2PrOH), 1-butanol (1BuOH), 1-pentanol (1PeOH), 1-octanol (1OcOH), and water (W). Three analyzed datasets are denoted as (A) [59,60], (B) [61], and (C)–this work. The last column comprises the values of the enthalpy–entropy compensation factor computed according to Equation (2).

Solvents	H^int^ [kcal/mol]	S^int^ [cal/mol/K)	R^2^	χ
MeOH ^(A)^	−23.09	69.80	0.996	0.64
EtOH ^(A)^	−24.91	72.07	0.997	0.66
1PrOH ^(A)^	−26.14	74.82	0.999	0.67
2PrOH ^(A)^	−29.32	84.01	0.997	0.69
W ^(A)^	−17.30	46.73	0.995	0.57
EtOH ^(B)^	7.02	−33.77	1.000	0.35
W ^(B)^	5.48	−24.99	0.967	0.29
MeOH ^(C)^	−22.77	68.54	0.994	0.63
EtOH ^(C)^	−26.17	76.03	0.984	0.67
1PrOH ^(C)^	−26.87	76.80	0.986	0.67
2PrOH ^(C)^	−31.22	89.90	0.980	0.70
1BuOH ^(C)^	−32.44	91.39	0.980	0.71
1PeOH ^(C)^	−40.38	117.41	0.988	0.75
1OcOH ^(C)^	−38.76	111.22	0.993	0.75

## Data Availability

All data supporting the reported results are available on request from the corresponding author.

## Supplementary Materials

The following supporting information can be downloaded at: https://www.mdpi.com/article/10.3390/molecules27165274/s1, Table S1: The solubility values of coumarin (CAS: 91-64-5) expressed as molar fractions (×10^3^) along with the standard deviation values (*n* = 3), Table S2: Statistical characteristics of linear regressions exemplified in Figure 8 and used for back-computations of solubility using COSMO-RS-DARE approach, Table S3: Collection of molecular descriptors used for determination of linear regression.
