# Supplementary material for: Application of COSMO-RS-DARE as a Tool for Testing Consistency of Solubility Data: Case of Coumarin in Neat Alcohols
Source: Molecules. 2022 Aug 18;27(16):5274. doi: 10.3390/molecules27165274 (PMC9413568; doi:10.3390/molecules27165274)
Supplement: Supplementary file 1 [file molecules-27-05274-s001.zip › molecules-1852636-supplementary.pdf]

Supplementary Materials

Application of COSMO-RS-DARE as a tool for testing consistency of solubility data: case of coumarin in neat alcohols

Piotr Cysewski, Tomasz Jeliński and Maciej Przybyłek

Department of Physical Chemistry, Pharmacy Faculty, Collegium Medicum of Bydgoszcz, Nicolaus Copernicus University in Toruń, Kurpińskiego 5, 85-950 Bydgoszcz, Poland

Table S1. The solubility values of coumarin (CAS: 91-64-5) expressed as molar fractions (×10^3) along with the standard deviation values (*n* = 3).

Solvent	Temperature [°C]			
	25	30	35	40
methanol, MeOH (CAS: 67-56-1)	77.01 ± 4.68	127.52 ± 5.07	196.63 ± 7.30	286.50 ± 6.20
ethanol, EtOH (CAS: 64-17-5)	49.43 ± 2.59	90.16 ± 5.57	142.45 ± 5.12	210.96 ± 6.24
1-propanol, 1PrOH (CAS: 71-23-8)	41.02 ± 0.72	74.27 ± 2.42	121.65 ± 5.97	181.10 ± 4.99
2-propanol, 2PrOH (CAS: 67-63-0)	30.17 ± 0.60	60.35 ± 1.81	101.22 ± 4.59	154.88 ± 5.76
1-butanol, 1BuOH (CAS: 71-36-3)	16.95 ± 1.09	34.87 ± 1.34	60.64 ± 0.77	94.59 ± 2.12
1-pentanol, 1PeOH (CAS: 71-41-0)	19.66 ± 0.28	42.95 ± 1.59	77.44 ± 0.55	126.01 ± 7.64
1-octanol, 1OcOH (CAS: 111-87-5)	19.18 ± 0.67	37.37 ± 1.24	67.23 ± 2.91	104.05 ± 0.71

Table S2. Statistical characteristics of linear regressions exemplified in Figure 8 and used for back-computations of solubility using COSMO-RS-DARE approach.

T[°C]	a±SD	b±SD	P	R^2_{adj}	F
**SpMin2_Bhm**					
25	-2.67±0.24	-1.18±0.31	<0.001	0.952	119.37
30	-2.44±0.20	-0.87±0.26	<0.001	0.960	143.82
35	-2.25±0.21	-0.66±0.26	<0.001	0.952	120.39
40	-2.12±0.21	-0.48±0.27	=0.001	0.941	97.395
**R_a**					
25	0.55±0.06	-12.47±0.90	<0.001	0.931	81.683
30	0.51±0.06	-11.20±0.82	<0.001	0.932	83.039
35	0.47±0.05	-10.17±0.80	<0.001	0.923	73.298
40	0.44±0.06	-9.42±0.83	=0.001	0.908	60.016

Table S3. Collection of molecular descriptors used for determination of linear regression. The Hansen solubility space distance, R_a, was calculated according to eq. 3 based on the Hansen solubility parameters (δ_d, δ_p ,δ_{HB}). The last column compiles the values of SpMin2_Bhm of considered alcohols.

Compound	δ_d	δ_p	δ_{HB}	R_a	SpMin2_Bhm
methanol	15.1	12.3	22.3	18.42	0.42
ethanol	15.8	8.8	19.4	15.67	0.91
1-propanol	16.0	6.8	17.4	14.53	1.21
2-propanol	15.8	6.1	16.4	14.34	1.25
1-butanol	16.0	5.7	15.8	13.89	1.41
1-pentanol	15.9	5.9	13.9	12.75	1.54
1-octanol	16.0	5.0	11.9	12.14	1.74
coumarin	20.0	12.5	6.7	-	-
